# WHITE MATTER INTEGRITY UNDERLYING SUBSYNDROMAL DEPRESSION SYMPTOMS IN DEMENTIA CAREGIVERS

**DOI:** 10.1093/geroni/igz038.3300

**Published:** 2019-11-08

**Authors:** Stephen Smagula, Tales Santini, Sarah Stahl, Tamer Ibrahim, Charles Reynolds, Richard Schulz, Layla Banihashemi, Liang Zhan

**Affiliations:** 1 Department of Psychiatry, School of Medicine, University of Pittsburgh, Pennsylvania, United States; 2 University of Pittsburgh, Pittsburgh, Pennsylvania, United States

## Abstract

Past research shows that major depression is associated with lower white matter integrity in fronto-limbic and other areas. But it is not known whether the integrity of these white matter connections is associated with subsyndromal depression symptoms, a marker of risk for major depression, in family dementia caregivers (dCGs) who reported stress. If specific aspects of white matter integrity are related to depression symptoms in this high-risk group, this could provide a biomarker of vulnerability or target for treatment. Participants included 41 dCGs (average age=69, standard deviation=6.4), who underwent a 7 Tesla 64-direction (12-minute) diffusion-weighted imaging sequence. Analyses compared dCGs with (n=20) and without (n=21) subsyndromal depression symptoms (nine-item Patient Health Questionnaire scores ≥5). Using fractional anisotropy (FA), we assessed differences in the integrity of 11 white matter aspects implicated in prior studies of major depression. We found that caregivers with subsyndromal depression had lower FA in tracts connecting to the posterior cingulate cortex (Cohen’s D=-0.9, p-value=0.006, FDR=0.03) and in white matter connecting the dorsolateral prefrontal cortex with the rostral cingulate (Cohen’s D=-1.2, p-value=0.0005, FDR=0.006). Thus, differences in the integrity of white matter (and related functions) reaching the posterior cingulate (autobiographical memory/planning) and connecting dorsolateral prefrontal and rostral cingulate regions (emotion re-appraisal) may contribute to depression vulnerability in dCGs. These observations require contextualizing further (e.g., assessing roles of depression history and other risk factors) for their meaning to be fully elucidated. Potentially, relationships between known risk factors (e.g., subjective stress) and depression emerge from or drive changes in white matter.

